# LATENT CLASSES OF FAMILY CAREGIVERS OF PATIENTS RECEIVING PALLIATIVE ONCOLOGY

**DOI:** 10.1093/geroni/igae098.0161

**Published:** 2024-12-31

**Authors:** Todd Becker, Kyle Pitzer, Elizabeth Bryant, Karla Washington

**Affiliations:** Washington University School of Medicine in St. Louis, St. Louis, Missouri, United States; Washington University in St. Louis, St. Louis, Missouri, United States; Washington University in St. Louis, St. Louis, Missouri, United States; Washington University in St. Louis, St. Louis, Missouri, United States

## Abstract

Family caregivers (FCGs) are recognized as the “invisible backbone” of cancer care, where the national median age of new diagnosis is 66 years. Although research on caregiver retention has prioritized examination of the demands placed on FCGs, research on the resources mitigating or exacerbating these demands is limited. Understanding this interplay is key to supporting FCGs’ continued partnership in care provision. Drawing on the job demands–resources model, we aimed to identify latent classes of FCGs of patients receiving palliative oncology by exploring how caregiving burden and resources overlap. As part of an ongoing multisite clinical trial on problem-solving therapy, we recruited 352 FCGs of patients receiving outpatient palliative care. We collected baseline data through established measures on FCGs’ resources (area deprivation index, disposable income, self-rated health, formal education) and caregiving demands (caregiving distance, number of work-related changes, frequency of caregiving). We, then, used these grouping variables to identify latent classes of FCGs. Results indicated that a four-class model provided the best fit (AIC = 5,337.97, BIC = 5,658.66, entropy =.70). These four classes corresponded to shifting combinations of resources and demands: low resources–low demands (12%), low resources–high demands (31%), high resources–low demands (17%), and high resources–high demands (40%). To our knowledge, these results provide the first assessment of distinguishable subgroups of FCGs of patients receiving outpatient palliative oncology by demands and resources. Identification of these subgroups represents a crucial first step toward developing and refining supportive caregiver interventions to facilitate well-being and retention in service of patient care.

